# Analysis of the Group of Pediatric Patients With Relapsing-Remitting Multiple Sclerosis: Data From the Czech National Registry

**DOI:** 10.3389/fneur.2022.851426

**Published:** 2022-04-18

**Authors:** Martin Vališ, Zbyšek Pavelek, Michal Novotný, Blanka Klímová, Jana Šarláková, Simona Halúsková, Marek Peterka, Ivana Štětkárová, Pavel Štourač, Jan Mareš, Pavel Hradílek, Radek Ampapa, Marta Vachová, Eva Recmanová, Eva Meluzínová

**Affiliations:** ^1^Department of Neurology, University Hospital Hradec Kralove, Hradec Kralove, Czechia; ^2^Department of Neurology, Faculty of Medicine, University Hospital Plzen, Charles University, Plzen, Czechia; ^3^Third Faculty of Medicine, Charles University and Hospital Kralovské Vinohrady, Charles University, Prague, Czechia; ^4^Department of Neurology, University Hospital, Masaryk University, Brno, Czechia; ^5^Department of Neurology, Faculty of Medicine, Palacky University and University Hospital Olomouc, Olomouc, Czechia; ^6^Clinic of Neurology, University Hospital Ostrava, Ostrava, Czechia; ^7^Department of Neurology, Hospital of Jihlava, Jihlava, Czechia; ^8^Department of Neurology, KZ a.s., Hospital Teplice, Teplice, Czechia; ^9^Department of Neurology, Tomas Bata Regional Hospital, Zlín, Czechia; ^10^Department of Neurology, Second Faculty of Medicine, Charles University, Prague, Czechia

**Keywords:** pediatric multiple sclerosis, relapsing-remitting form, disease-modifying drugs, interferon beta-a, glatiramer acetate

## Abstract

**Importance:**

Multiple sclerosis can also affect children. Approximately 3–10% of patients develop multiple sclerosis before the age of 16.

**Objective:**

The aim of this analysis is to describe the characteristics of pediatric patients with multiple sclerosis who started their treatment with disease-modifying drugs in 2013–2020, with data obtained from the Czech National Registry of patients with multiple sclerosis.

**Design and Setting:**

A method of retrospective analysis conducted with 134 pediatric patients with multiple sclerosis was used.

**Results:**

The findings reveal that the mean age at the date of the introduction of the first disease-modifying drugs treatment is 15.89 years, and gender does not play any role. In addition, moderate (51.6%) and mild (45.2%) relapses are predominant in these young patients. Seventy five percent of patients will not experience a confirmed progression of the expanded disability status scale within 54.7 months from starting the treatment. Furthermore, the results confirm that the first-choice treatment is interferon beta-a and glatiramer acetate, which is common for adult patients. However, some factors, such as a low efficacy or a lack of tolerance may impact on treatment discontinuation in children.

**Conclusion:**

More research should be performed on novel disease-modifying drugs for this target group.

## Introduction

Multiple sclerosis (MS) is a chronic autoimmune inflammatory disease associated with pathological processes in the central nervous system affecting mostly younger adults (20–40 years of age) (1, 2). However, multiple sclerosis can also affect children. Approximately 3–10% of patients develop MS before the age of 16, and in 1% it is even before the age of 10. Research shows an incidence of pediatric MS between 0.13 and 0.66 per 100,000 children per year (3, 4). The relapsing-remitting form of MS occurs in 98% of patients (1). Compared to adults, the pediatric form has more frequent relapses, more rapid lesion expansion early in the disease with more pronounced aspects of inflammation, worse cognitive decline, and worse physical disability over a longer time frame (5).

An important characteristics of childhood MS, compared to adults, is the longer time between disease onset and disability accumulation. This suggests that children have a greater ability to compensate for inflammatory brain damage despite high rates of relapse. The ARR (annualized relapse rate) is used to characterize the average number of relapses per patient per year. In contrast, the transition time from mild to severe disability, which is approximately 10 years, is similar in children and adults and is thought to be mainly due to neurodegeneration (5, 6). A scoring system called the expanded disability status scale (EDSS) is used to quantify disability in MS patients.

Early and correct diagnosis connected with prompt initiation of appropriate treatment is of paramount importance for improved long-term prognosis of the patient, including lower rates of relapse and worsening disability, as evidenced by recent publications on pediatric-onset multiple sclerosis (7, 8) and also pediatric guidelines for MS therapy recommend starting treatment as early as possible to prevent disease (7, 8). As in the treatment of adults, the drugs of choice for pediatric patients are disease-modifying drugs (DMDs), which target the peripheral immune system to reduce the risk of MS relapses. Currently, only two molecules from the DMD family are approved (FDA/EMA) for pediatric use and have been studied in Phase III clinical trials. These are fingolimod and teriflunomide. However, it is quite common that interferon-β and glatiramer acetate are also used in clinical practice. Clinical practice and recent publications show that an increasing number of pediatric patients are being treated with dimethyl fumarate or natalizumab. However, it should be noted that a number of phase II and III trials are currently underway to evaluate the efficacy and safety of unapproved molecules in pediatric MS patients (1, 9–11). The treatment of pediatric patients is strictly indicated in specialized centers and is mostly based on recommended treatment protocols for adult patients (12).

The aim of this analysis is to describe the characteristics of pediatric patients with multiple sclerosis who started their treatment with disease-modifying drugs in 2013–2020, with data obtained from the Czech National Registry of MS patients (ReMuS).

## Materials and Methods

### Design

This was a retrospective analysis of the Czech National Registry of MS patients ReMuS. The details about the ReMuS were described in another research study, please consult Pavelek et al. (2).

### Analyzed Population

For the purpose of this analysis, a group of patients with multiple sclerosis who started their first treatment with DMD drugs between 2013 and 2020 and were <18 years old on the date of starting this therapy was selected from the ReMuS registry. Three time points were defined throughout the follow-up period: the BL (first line; treatment start of a 2-yr follow-up); M12 (follow-up at 12 months after the introduction of treatment); and M24 (follow-up at 24 months after the introduction of treatment). Patients were divided into three groups: group A are patients who started their first DMD therapy between 2013 and 2018 and were younger than 16 years (i.e., had at least 2 years of follow-up before the age of 18). Group B are patients who received their first DMD therapy between 2013 and 2018 and were both older than 16 years and younger than 18 years (i.e., had reached adulthood during the 2-year follow-up period). Group C are patients who started treatment in 2019 or 2020 and therefore do not have the full two-year follow-up period covered.

### Endpoints

The aim of this analysis is to describe the characteristics of pediatric patients with multiple sclerosis who started their treatment with disease-modifying drugs between 2013 and 2020. The molecules monitored are as follows: (a) state-approved molecules (i.e., in the Czech Republic) for pediatric use: fingolimod and teriflunomide; (b) drugs approved for use in adults with MS: interferon beta-1-a, peginterferon beta-1-a, interferon beta-1-b, glatiramer acetate; dimethyl fumarate; natalizumab; (c) an off-label molecule: rituximab. In addition to the basic demographic data, the following data were analyzed. In terms of relapses that were observed in group A and B, these were ARR and severity of relapses and time to the first relapse in the treatment setting.

In this analysis the EDSS obtained may not have been captured accurately at the three time points (BL, M12, M24) and therefore had to be extrapolated in the follow-up period. Disability was monitored only for group A, and from several perspectives: changes in EDSS, including absolute changes according to their magnitude, were monitored; time to the confirmed EDSS progression was analyzed; and the relationship between disability and severity of the first relapse was determined. For the purpose of calculating the time to the confirmed progression, the EDSS at the date of the introduction of the first DMD treatment was rounded to the nearest valid EDSS value. For example, the EDSS on the day of treatment onset was calculated as follows: the nearest measured EDSS before the date of the onset of the first DMD treatment and the nearest measured EDSS after the date of the onset of the first DMD treatment were connected using a straight line. Subsequently, the EDSS at the treatment start date is estimated using this straight line. In addition, a check was also made to ensure that the EDSS measurement was not too far from the observation date (e.g., from the start of the first DMD treatment). If there was more than a year between EDSS measurements, then the EDSS that was within 90 days (inclusive) of the observation date (for example, from the start of the 1st DMD treatment) was used. If no such value was available, the EDSS was considered missing and the value was therefore not further processed. The confirmed progression is defined as a change in EDSS of 1.5 points or more for patients with a first line EDSS of 0 and 1 point for patients with a first line EDSS of 1 or more if the change persists for at least 6 months. The analysis was performed only for group A. Time to the confirmed progression was calculated using the Kaplan-Meier estimator. As the confirmed progression did not occur in a sufficient number of patients to estimate the median time to confirmed progression, the upper quartile was estimated.

Regarding the therapy, the initial DMD therapy was analyzed in detail according to the active molecules. It was also observed how many patients discontinued the first DMD therapy and after how long. Adverse effects and reported reasons for changing/terminating the first DMD therapy were monitored simultaneously. Subsequently, changes in lines of therapy in patients who discontinued their first DMD therapy were also monitored.

### Statistical Analysis

The aim of this analysis is to describe the characteristics of pediatric patients with multiple sclerosis who started their treatment with disease-modifying drugs or other off-label drugs (fingolimod, teriflunomide, interferon beta-1-a, peginterferon beta-1-a, interferon beta-1-b, glatiramer acetate, dimethyl fumarate, natalizumab, rituximab, alemtuzumab, cladribine or ocrelizumab.) between 2013 and 2020. In addition to the basic demographic data, the following data were analyzed. In terms of relapses that were observed in group A and B, these were ARR and severity of relapses and time to the first relapse in the treatment setting. Disability was monitored only for group A, and from several perspectives: changes in EDSS, including absolute changes according to their magnitude, were monitored; time to the confirmed EDSS progression was analyzed; and the relationship between disability and severity of the first relapse was determined. Regarding the therapy, the initial DMD therapy was analyzed in detail according to the active molecules. It was also observed how many patients discontinued the first DMD therapy and after how long. Adverse effects and reported reasons for changing/terminating the first DMD therapy were monitored simultaneously. Subsequently, changes in lines of therapy in patients who discontinued their first DMD therapy were also monitored.

## Results

### Demography of the Analyzed Groups

The analysis included 134 pediatric patients who were divided into 3 groups (see Methods–Study population; Table 1). There were 44 patients in group A (yellow), 57 patients in group B (green), and 33 patients in group C (blue). Of the 134 patients studied, 36 (26.9%) were boys and 98 (73.1%) were girls. A more detailed analysis by group revealed that 29.5% of boys and 70.5% of girls were in group A, with a mean age of 14.6 and 14.1 years at the time of the introduction of the first DMD treatment. In group B, there were 24.6% of boys and 75.4% of girls with a mean age of 17 and 17.2 years at the introduction of the first DMD treatment. Group C included 27.3% of boys and 72.7% of girls who were 15.7 and 16.1 years old, respectively, at the time of the introduction of the first DMD treatment.

**Table 1:** Demographic characteristics of the pediatric population at the time of the introduction of DMD treatment (by age and by year).

**Age**	**Introduction of the first DMD treatment**								**Total**
	**2013**	**2014**	**2015**	**2016**	**2017**	**2018**	**2019**	**2020**	
8	0	1	1	0	0	0	0	0	2
9	0	0	1	0	0	0	0	0	1
10	0	0	1	0	1	0	0	0	2
11	0	0	0	0	0	0	0	1	1
12	0	0	1	0	1	1	1	0	4
13	0	0	2	1	0	1	0	2	6
14	1	1	2	2	4	0	1	1	12
15	6	5	3	2	5	1	4	4	30
16	6	2	3	3	4	6	7	4	35
17	6	6	2	7	5	7	4	4	41
Total	19	15	16	15	20	16	17	16	134

*Green colour - patients on injectables; Blue colour - patients on newer DMT; Yellow colour - patient without DMT*.

### Relapses

The average annual relapse rate was analyzed only for groups A and B, as the entire 2-year period was monitored and the relapse rates can therefore be compared (Table 2). During the follow-up period, 27 (61.4%) patients in group A did not experience any relapse, while in group B, 31 (54.4%) patients did not have any relapse. Time to the first relapse was calculated as the difference between the date of the first relapse after the introduction of the follow-up and the date of the introduction of the follow-up (i.e., the introduction of the first DMD treatment) and calculated using the Kaplan-Meier estimator. A significant difference (*p* = 0.035) was found in the data obtained, with half of the boys relapsing within 42.6 months, whereas half of the girls relapsed within 26.7 months from the introduction of the first DMD treatment.

**Table 2:** Average annual relapse rate (AAR) and the characteristics of relapses during the follow-up period.

**Group**	**Number of patients**	**ARR**	**Relapses in the monitored period (24 months)**			
			**Total**	**Mild**	**Moderate**	**Severe**
A	44	0.352	31	45.2%	51.6%	3.2%
B	57	0.404	46	56.5%	43.5%	0.0%

### Disability

The disability was analyzed only in group A. The patients for whom the EDSS value could not be determined at the time of the start of the follow-up were excluded from the disability analysis (*N* = 2). The mean EDSS was 1.47 at BL, 1.5 at M12 and 1.48 at M24.

It can be assumed that 75% of patients will not experience the confirmed EDSS progression within 54.7 months from the introduction of treatment. In addition, the relationship between the first relapse and disability was also examined in group A (Table 3). Of the 44 patients studied, 42 patients were further considered for the analysis, as 2 patients had not relapsed before the recorded introduction of treatment.

**Table 3:** Relationship between the first relapse and disability (Group A).

**Disability of the first relapse before the introduction of DMD treatment**	**Number of patients**	**EDSS at the time of the introduction of the first DMD treatment**				**EDSS in M12**				**EDSS in M24**			
		**Average**	**SD**	**Median**	**NA**	**Average**	**SD**	**Median**	**NA**	**Average**	**SD**	**Median**	**NA**
Mild	12	1.46	0.891	1.5	0	1.65	0.906	1.8	0	1.52	0.842	1.5	0
Moderate	30	1.47	0.675	1.5	0	1.43	0.807	1.5	1	1.46	0.608	1.5	0

### Therapy

For all groups, the first DMD treatment was analyzed by looking at the number of patients in each group relative to the active molecule and the mean time to the treatment completion (Table 4). The change in the strength of the drug was also included in the DMD drug discontinuation and change. If we do not consider this change in the strength of the drug as a discontinuation of the therapy, then 77% of patients in group A, 68% of patients in group B and 24% of patients in group C would have discontinued the therapy. The mean time to the completion of the first DMD treatment would be 27.2 months in group A, 20.2 months in group B and 7.4 months in group C. The uncompleted therapies were not included in the mean time to the completion.

**Table 4:** Overview of the first DMD treatment for all groups by active molecules.

**Group**	**The first DMD treatment**	**Number of patients**	**Completed**		
			**Number**	**Percentage**	**Mean time to the completion of treatment (months)**
A	Glatiramer-acetate 20 MG	20	19	95%	24.83
	Interferon beta-1a 44 MCG	9	7	78%	15.58
	Glatiramer-acetate 40 MG	7	5	71%	19.49
	Interferon beta-1a 30 MCG	4	4	100%	38.85
	Interferon beta-1a 22 MCG	2	2	100%	24.25
	Peginterferon beta-1a	1	1	100%	13.34
	Natalizumab	1	1	100%	36.17
B	Glatiramer-acetate 40 MG	17	8	47%	20.33
	Interferon beta-1a 44 MCG	14	11	79%	23.09
	Glatiramer-acetate 20 MG	13	13	100%	11.42
	Interferon beta-1a	6	4	67%	28.62
	Peginterferon beta-1a	4	4	100%	13.4
	Interferon beta-1b	1	1	100%	16.43
	Fingolimod	1	1	100%	27.63
	Natalizumab	1	-	0%	-
C	Interferon beta-1a 44 MCG	10	2	20%	233%
	Glatiramer-acetate 40 MG	9	2	22%	830%
	Peginterferon beta-1a	7	2	29%	5.59
	Interferon beta-1a	3	-	0%	-
	Glatiramer-acetate 20 MG	2	2	100%	15.82
	Teriflunomid	1	-	0%	-
	Interferon beta-1a 22 MCG	1	1	100%	6.41
A		44	39	89%	23.89
B		57	42	74%	18.5
C		33	9	27%	7.83

For the patients who were not given an identical drug of different intensity after the discontinuation of the first line DMD, the reasons for stopping the treatment were monitored. There were a total of 28 such patients in group A, of whom 21 discontinued due to efficacy, 5 due to poor tolerability of the drug, 1 due to adherence/comfort, and one due to an unknown reason. The mean time to discontinuation was 22.4 months for discontinuation due to efficacy and 18.1 months for tolerability. In group B, the most common reasons for discontinuation of the first DMD treatment were efficacy (21 patients) and tolerability (7 patients). The mean time to the treatment discontinuation was 19.8 months for efficacy and 18.3 months for tolerability. Only one adverse event (immediate post-injection reaction) was recorded, and this was for group B and glatiramer acetate 20 MG. The treatment was discontinued after less than three (specifically 2.76) months.

Changes in the treatment lines in the patients who completed their first DMD treatment are analyzed only in group A, given that 89% of patients in this group discontinued their first DMD treatment (Table 5). The first DMD drug was a first line drug for 38 patients. Of these, 22 patients switched to another first line drug after completing this treatment, 15 patients switched to a second line drug after completing the first DMD treatment, and 1 patient did not start any additional DMD drug.

**Table 5:** Summary of changes in the treatment lines in patients who have completed their first DMD treatment.

**1. DMD**	**2. DMD**	**Number**	**Percentage**	**Average delay (months)**
First line	First line	11	28.21%	0.06
First line	Only the change in in the strength of the drug	11	28.21%	0.03
First line	Escalation line	15	38.46%	0.55
First line	Unprescribed	1	2.56%	NA
Escalation line	Escalation line	1	2.56%	2
Total		39	100.00%	

## Discussion

Research studies reveal that ~3–5% of all individuals diagnosed with MS will experience their first attack before the age of 16 (13–15). Our analysis expands these findings, showing that of the 134 pediatric patients studied, 44 were aged 0–15 years and 57 were aged 16–17 years. Even the two youngest patients started their first DMD at the age of eight in 2014 and 2015. One nine-year-old and one ten-year-old patient also started their first DMD treatment in 2015. However, the mean age at the date of the introduction of the first DMD treatment is 15.89 years, and gender does not play a role (girls/boys −15.90/15.80).

Childhood MS is considered to be a highly active form with more frequent relapses (2–3 times more frequent relapses than adults with early-onset MS), lesions early in the disease, and worse cognitive and physical impairment earlier in life (16, 17). Research shows that the increased frequency of relapses persists for about the first 6 years of the disease (18). Interestingly, children recover from relapses faster than adults, on average 4 weeks compared to 6–8 weeks for adults (19). Our analysis shows that in group A, 31 relapses were recorded in the two-year follow-up period, i.e., the ARR is 0.352 relapses/year. This means one relapse per year for approximately one in three patients. Moderate (51.6%) and mild (45.2%) relapses were predominant in the study period. In group B, 46 relapses were recorded in the two-year follow-up period, i.e., ARR is 0.404 relapses/year and mild (56.5%) and moderate (43.5%) relapses were prevalent. Thus, it can be said that the frequency of relapses is higher in group B than in group A, but in this latter group they are more severe in nature.

In pediatric MS patients, there is a trend that the disability occurs at a younger age (6, 20). At the time of the start of the follow-up period, the mean EDSS for Group A was 1.47 and appeared not to have changed significantly over the follow-up period. On the basis of the available data, we assume that 75% of patients will not experience a confirmed progression of EDSS within 54.7 months of starting the treatment.

It is quite common clinical practice that DMDs that are registered for adult patients are prescribed for pediatric patients. The first-choice treatment is interferon beta-a and glatiramer acetate. This was confirmed in our analysis of the ReMuS registry data. For example, in group A, glatiramer acetate (45.5% of patients) and interferon beta-a (20.5% of pediatric patients) were the most frequent first DMD drugs. The safety and efficacy of these drugs have been demonstrated in small retrospective studies, case studies and unblinded controlled trials (21, 22). However, these molecules still need to have an official approval by state authorities. A lack of tolerance or continued disease progression despite these therapies may require the use of other therapies.

In our analysis, 77% of patients in group A, 68% of patients in group B, and 24% of patients in group C discontinued the first DMD treatment. The mean time to the discontinuation of the first DMD treatment would be 27.2 months in group A, 20.2 months in group B and 7.4 months in group C. In group A, 21 patients discontinued treatment due to low efficacy, and the mean time to discontinuation for this reason was 22.4 months. In group B, low efficacy was also the most common reason for discontinuation (21 patients) and the mean time to discontinuation for the same reason was 19.8 months. However, as mentioned above, none of the pediatric patients should be left untreated; 28.21% (11 patients) were switched to another first-choice medication, 28.21% (11 patients) had a change in strength of the drug, and 38.46% (15 patients) were put on an escalation line.

**Figure 1:**
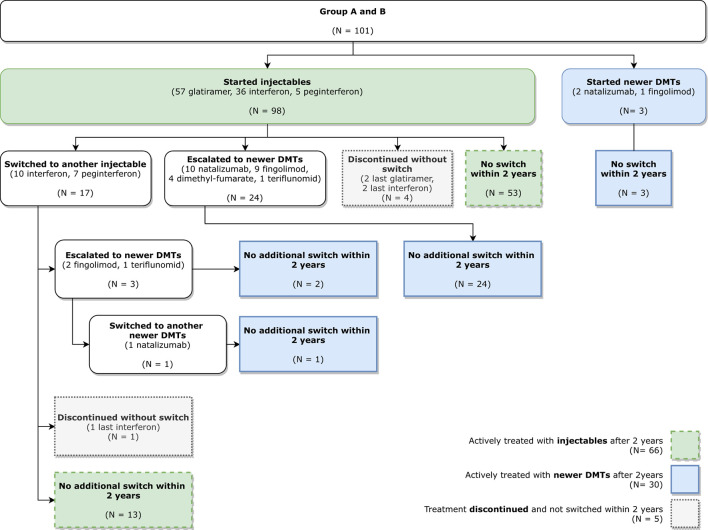
Patient disease-modifying therapy (DMT) pathway.

As Figure 1 below shows, out of 101 patients, 56 patients (53 with first line drug treatment and 3 with second line drug treatment) receive the same treatment after 2 years = 55.45%. In addition, out of 98 patients who started on first line drug treatment, 27 (27.55%) patients switched to the second line drug treatment within 24 months. Similar findings were confirmed by a UK study (12).

In conclusion, it should be stated that this certainly does not exhaust all treatment options and other therapies for MS, including dimethyl fumarate or rituximab that are currently under investigation in clinical trials for the treatment of pediatric MS (5, 23). For example, in a cohort study comparing the first-line treatment with novel DMDs in children with MS, the newer DMDs provided better disease control, but further studies are still needed, particularly on safety (24). For completeness, it can be added that due to the highly active nature of pediatric MS, some authors recommend starting the treatment with novel DMTs with expected higher efficacy (17).

## Data Availability Statement

The raw data supporting the conclusions of this article will be made available by the authors, without undue reservation.

## Ethics Statement

Ethical review and approval was not required for the study on human participants in accordance with the local legislation and institutional requirements. Written informed consent to participate in this study was provided by the participants' legal guardian/next of kin.

## Author Contributions

All authors have equally contributed to drafting, writing, and revising this article. They all have approved its final version.

## Funding

This article was supported by the Ministry of Health of the Czech Republic (DRO – UHHK 00179906), Charles University, and Czech Republic (PROGRES Q40).

## Conflict of Interest

MVac was employed by KZ a.s., Hospital Teplice. The remaining authors declare that the research was conducted in the absence of any commercial or financial relationships that could be construed as a potential conflict of interest.

## Publisher's Note

All claims expressed in this article are solely those of the authors and do not necessarily represent those of their affiliated organizations, or those of the publisher, the editors and the reviewers. Any product that may be evaluated in this article, or claim that may be made by its manufacturer, is not guaranteed or endorsed by the publisher.
